# Body composition assessment of people with overweight/obesity with a simplified magnetic resonance imaging method

**DOI:** 10.1038/s41598-023-37245-3

**Published:** 2023-07-10

**Authors:** Yoann Pereira, Monique Mendelson, Mathieu Marillier, Abdallah Ghaith, Samuel Verges, Anna Borowik, Nicolas Vuillerme, François Estève, Patrice Flore

**Affiliations:** 1 Univ. Grenoble Alpes, Inserm, CHU Grenoble Alpes, HP2, 38000 Grenoble, France; 2 Univ. Grenoble Alpes, AGEIS, Grenoble, France; 3 Institut Universitaire de France, Paris, France; 4 Inserm – UA07 – Rayonnement Synchrotron pour la Recherche Biomédicale (STROBE) ID17 Installation Européenne du Rayonnement Synchrotron (ESRF), Grenoble, France; 5 CLUNI, CHU Grenoble Alpes, Grenoble, France; 6 UM Sports Pathologies, Hôpital Sud, Avenue Kimberley, CS 90338, 38434 Echirolles-Cedex, France

**Keywords:** Imaging, Anatomy, Predictive markers

## Abstract

To develop a simplified magnetic resonance imaging method (MRI) to assess total adipose tissue (AT) and adipose tissue free mass (ATFM) from three single MRI slices in people with overweight/obesity in order to implement body composition follow-up in a clinical research setting. Body composition of 310 participants (70 women and 240 men, age: 50.8 ± 10.6 years, BMI: 31.3 ± 5.6 kg.m^−2^) was assessed with 3 single slices (T6-T7, L4-L5 and at mid-thigh) MRI. Multiple regression analysis was used to develop equations predicting AT and ATFM from these three single slices. Then we implemented a longitudinal phase consisting in a 2-month exercise training program during which we tested the sensitivity of these equations in a subgroup of participants with overweight/obesity (n = 79) by comparing the exercise-induced variations between predicted and measured AT and ATFM. The following equations: total AT = − 12.74105 + (0.02919 × age) + (4.27634 × sex (M = 0, F = 1)) + (0.22008 × weight) + (26.92234 × AT T6-T7) + (23.70142 × AT L4-L5) + (37.94739 × AT mid-thigh) and total ATFM = − 33.10721 + (− 0.02363 × age) + (− 3.58052 × sex (M = 0, F = 1)) + (30.02252 × height) + (0.08549 × weight) + (11.36859 × ATFM T6-T7) + (27.82244 × ATFM L4-L5) + (58.62648 × ATFM mid-thigh) showed an excellent prediction (adjusted R^2^ = 97.2% and R^2^ = 92.5%; CCC = 0.986 and 0.962, respectively). There was no significant difference between predicted and measured methods regarding the AT variations (− 0.07 ± 2.02 kg, p = 0.70) and the ATFM variations (0.16 ± 2.41 kg, p = 0.49) induced by 2-months of exercise training. This simplified method allows a fully accurate assessment of the body composition of people with obesity in less than 20 min (10 min for images acquisition and analysis, respectively), useful for a follow-up.

## Introduction

Obesity is a major public health issue^1^. The consequences are numerous and include cardiovascular diseases, metabolic syndrome, non-alcoholic fatty liver disease and obstructive sleep apnea^2–5^. These adverse outcomes increasingly affect adults and children^1,6,7^. The assessment of adipose tissue (AT) and adipose tissue free mass (ATFM; ATFM = total body mass − AT)^8^ are essential for the evaluation and management of obesity^9–15^. Body composition is correlated with cardiometabolic risk^2,3,5^ and is a strong marker to assess the effects of an intervention including diet and/or exercise training^10,13,14,16^.

Magnetic resonance imaging (MRI) is the gold standard^16–21^ to quantify skeletal muscle and adipose tissue, and to assess its distribution. Owing to the relatively high cost of MRI^22^ and the elevated image-acquisition time attributed to multi-slice imaging^16^, a single slice at the abdomen is often used in various clinical settings^14,23–32^. However, this measure is not sensitive to interindividual morphological differences, in particular fat distribution (e.g. android or gynoid obesity)^33^.

Moreover, fat evaluation at the thoracic level T6-T7^34^ may represent a good assessment of thoracic obesity independently of sex^35^. Although visceral fat is correlated with obesity-related diseases^9,11,12,15,36^, it is important to analyze whole-body composition to thoroughly assess the effects of different interventions such as exercise and diet in clinical settings. A very interesting study showed that a single slice at L3 was the best compromise to assess total tissue volumes of whole-body skeletal muscle, VAT, and SAT^27^. However, the authors reported a lack of sensitivity to track changes of body composition probably because of lack of specificity of this slice. In addition, since over 50% of whole-body muscle content is located in the legs^27,37^, it appears appropriate to measure body composition in this area. A mid-thigh slice has been shown to reflect whole-body muscle mass in both obese^38^ and normal-weight individuals^27,38,39^. Moreover, Schweitzer et al.^27^ reported that for whole skeletal muscle volume, the optimal slice area was that obtained at mid-thigh and not the compromise, i.e. L3, and that during a follow-up with weight-loss changes, unsurprisingly, the area at L3 reflected changes in total VAT and SAT. These results lead the authors to conclude that areas at mid-thigh showed the best evidence to assess changes in total skeletal volume.

Taken together these results and ours suggest that the best way to quickly and accurately estimate and follow adipose tissue and adipose tissues free mass was to incorporate 3 single slices in a model.

The aim of this study was thus to build and validate equations allowing quick but accurate determination of total AT and ATFM from three single MRI slices at chest (T6-T7), abdomen (L4-L5) and mid-thigh levels in order to facilitate the clinical follow-up of people with overweight/obesity. To achieve this goal, we tested the concordance and agreement between the predictive and the measured body composition in a subgroup of people of both sexes with overweight and obesity. We also aimed to assess the sensitivity of equations to detect changes in body composition induced by an (aerobic) exercise training program. We hypothesized that body composition would be accurately assessed and followed up over time in people with overweight and obesity using predictive equations.

## Materials/subjects and method

### Subjects

Subjects (n = 310) were participants from a large prospective study conducted at the University Hospital of Grenoble Alps (HYPINT: NCT02642705); we measured and analyzed body composition using MRI in 70 women and 240 men aged 50.8 ± 10.6 years (range: 20–66 years) with a BMI of 31.3 ± 5.6 kg.m^−2^ (range 17.9–51.0) (Table 1). Thirty-eight participants had a BMI lower than 25 kg.m^−2^, 93 between 25 and 30 kg.m^−2^, and 179 greater than 30 kg.m^−2^.

**Table 1:** Demographics, total adipose tissue (AT), total adipose tissue free mass (ATFM) of total population, women and men with overweight/obesity, and trained subgroup with overweight/obesity.

	Total		Normal weight		Overweight		Obese		Trained group data	
	(n = 310)		Women (n = 9)	Men (n = 29)	Women (n = 14)	Men (n = 79)	Women (n = 47)	Men (n = 132)	Women (n = 20)	Men (n = 59)
	Mean ± SD	Range	Mean ± SD	Mean ± SD	Mean ± SD	Mean ± SD	Mean ± SD	Mean ± SD	Mean ± SD	Mean ± SD
Age, yr	50.8 ± 10.6	20–66	31.8 ± 11.0	44.8 ± 12.0	57.6 ± 5.91	54.3 ± 7.63	47.9 ± 9.85	51.8 ± 10.1	50.3 ± 8.80	53.2 ± 9.11
Weight, kg	93.0 ± 18.8	48.0–152.7	56.3 ± 4.5	68.6 ± 6.32	70.9 ± 3.29	87.9 ± 7.81	96.7 ± 18.7	104.9 ± 14.9	89.8 ± 20.0	97.4 ± 14.7
Height, m	1.72 ± 0.08	1.56–1.90	1.63 ± 0.03	1.74 ± 0.05	1.60 ± 0.02	1.76 ± 0.07	1.64 ± 0.06	1.74 ± 0.06	1.63 ± 0.05	1.75 ± 0.07
Body mass index, kg.m^-2^	31.3 ± 5.61	17.0–51.0	21.3 ± 1.29	22.5 ± 1.47	27.8 ± 0.86	28.4 ± 1.11	35.7 ± 4.8	34.5 ± 4.08	33.5 ± 5.50	31.8 ± 4.31
AT, kg										
Total AT mass	32.4 ± 13.3	3.69–85.6	13.34 ± 3.66	12.03 ± 5.23	29.9 ± 3.80	26.1 ± 3.77	46.4 ± 12.8	37.2 ± 10.2	42.3 ± 13.7	32.0 ± 8.84
AT T6-T7	0.20 ± 0.10	0.01–0.65	0.06 ± 0.02	0.07 ± 0.05	0.21 ± 0.06	0.16 ± 0.03	0.29 ± 0.11	0.24 ± 0.08	0.27 ± 0.11	0.19 ± 0.07
AT L4-L5	0.43 ± 0.15	0.07–0.97	0.14 ± 0.05	0.19 ± 0.08	0.35 ± 0.04	0.37 ± 0.06	0.53 ± 0.13	0.50 ± 0.12	0.50 ± 0.14	0.44 ± 0.11
AT mid-thigh	0.17 ± 0.10	0.01–0.59	0.11 ± 0.02	0.07 ± 0.02	0.19 ± 0.06	0.13 ± 0.04	0.31 ± 0.10	0.18 ± 0.08	0.29 ± 0.08	0.15 ± 0.07
ATFM, kg										
Total ATFM	62.3 ± 10.3	37.6–88.5	42.3 ± 1.75	59.75 ± 7.06	43.8 ± 3.40	63.7 ± 6.57	52.1 ± 6.4	68.9 ± 7.00	49.7 ± 6.57	66.7 ± 7.90
ATFM T6-T7	0.65 ± 0.10	0.39–0.88	0.44 ± 0.04	0.61 ± 0.06	0.48 ± 0.04	0.66 ± 0.06	0.56 ± 0.07	0.71 ± 0.07	0.54 ± 0.08	0.69 ± 0.07
ATFM L4-L5	0.33 ± 0.06	0.17–0.60	0.26 ± 0.02	0.30 ± 0.05	0.27 ± 0.04	0.33 ± 0.04	0.28 ± 0.04	0.37 ± 0.05	0.28 ± 0.05	0.35 ± 0.05
ATFM mid-thigh	0.36 ± 0.07	0.22–0.56	0.24 ± 0.02	0.33 ± 0.04	0.25 ± 0.01	0.36 ± 0.04	0.31 ± 0.04	0.41 ± 0.05	0.29 ± 0.04	0.39 ± 0.05

### Experimental design

We built equations in the total population (n = 310) and tested their validity in this population and in different BMI subgroup split into 2 parts according to sex (Table 1). Then we implemented a longitudinal phase consisting in a 2-month exercise training program during which we tested the sensitivity of these equations in a subgroup of participants with overweight/obesity (n = 79, 20 women, age: 52.7 ± 9.1 years; BMI: 32.1 ± 4.6 kg.m^−2^) by comparing the exercise-induced variations between predicted and measured AT and ATFM.

We used data from a completed clinical trial (NCT02642705) testing the cardiometabolic effects of different exercise training modalities (i.e. three 45-min sessions of ergocycle training per week for 2-months): (1) moderate intensity continuous training (MICT; n = 32) and (2) high intensity intermittent training (HIIT; n = 47). Body composition was assessed before (T0) and after the 2-month (T2) training period. Participants’ characteristics are summarized in Table 1.

The study was approved by the local ethics committee (Comité de Protection des Personnes Sud-Est V) and performed according to the Declaration of Helsinki. Participants were informed of the procedure and risks involved and gave their written consent prior to all assessments.

### Tissue measurement by magnetic resonance imaging (MRI)

MRI is a reference method for assessing body composition including AT, ATFM and its regional distribution, and particularly visceral adipose tissue^16,36^.

In order to determine full body composition (called measured body composition), the reference method includes 41 slices with a thickness of 10 mm spaced by 40 mm^16^. MRI data were acquired in 30 min for each participant on a General Electric Signa Advantage 1.5-T scanner (General Electric Medical Systems, Milwaukee, WI, USA). For the specific purpose of the present study (prediction of body composition), three single slices (thickness of 10 mm) were acquired in 10 min at 3 different localizations: T6-T7, L4-L5 and mid-thigh.

Mid-thigh level for the single slice acquisition was determined by computing half of the overall femoral length. In our medical department, we used our own equation (unpublished results) to measure the half-femoral length. In men, FL = (S − 67.76)/4.4 and in women, FL = (S—61.4) / 4.6 (with FL being the half-femoral length and S the stature). Indeed, statistical analysis of different populations shows that the stature can be derived from the femoral length^40^. Thus, half of the femoral length can be determined from the subject’s size. We compared the formula used in our medical department to those available in the literature^41^. The average difference of half-femoral length between our method and the one described in Trotter (1970) was only of 0.3 ± 0.26 cm in our population.

### Calculation of adipose tissue and adipose tissue free mass volumes

Each slice was analyzed using Matlab-based software (Matlab^®^, Mathworks, Inc.) developed by the radiology department of University Hospital of Grenoble Alps. The brightness level of tissue distinguished AT and ATFM, using the graphical interface of Matlab^®^. More specifically, we measured adipose tissue and calculated adipose tissue free mass by subtracting adipose tissue area to slice area. Moreover, determination of tissue area on a given MR image is performed by subjecting the data to various segmentation techniques. In our laboratory we have developed a computer software specifically designed for MR image analysis similar to that developed by Ross et al.^20^. The program features an interactive slice editor routine that allows for the verification of the segmentation result. This feature helps to assure that the area (cm^2^) values for the tissues of interest are accurately and reliably measured. Each slice was visually controlled to avoid an error in tissue type assignment, after histogram analysis to define a threshold for fat tissue segmentation. As our goal was to measure two types of tissues: adipose and adipose tissue free mass, we used a simplified method derived from the one developed by Ross et al.^16,17^. After threshold definition, the segmented image was compared to the MR image to correct wrong tissue type assignment when necessary. For instance, small isolated white dots occurring for instance when the slice concerned only a very small amount of isolated adipose tissue (so-called partial volume effect) were not considered in the AT amount. On the other side, weak MR signals occurring sometimes in the subcutaneous fat tissues were manually added to the AT. As initial double blinded initial tests did not result in significant differences, we considered that the AT amount measurement process was reliable.

To calculate the adipose tissue and lean tissue volumes in each slice, the program multiplies the number of pixels by the pixel surface (cm^2^) and the thickness (10 mm) of the slice. Whole-body adipose tissue and lean tissue volumes were calculated using the truncated pyramid method^16^. At last, the volume (in liters) of adipose tissue and lean tissue was converted to mass (kg) by multiplying the volumes of the assumed constant density of 0.92 for adipose tissue (kg.L^−1^)^42^ and 1.04 for lean tissue (kg.L^−1^)^38^.

In a preliminary internal laboratory study (n = 11), we showed a precision (test–retest) of 0.42% for total adipose tissue, 5.49% for the adipose tissue in T6-T7, 1.1% for the adipose tissue in L4-L5 and 1.2% for that in mid-thigh. The inter-observer variability was respectively 2.6%, 3.1%, 2.2% and 1.6%.

### Sensitivity of the prediction models to an exercise training program

We tested the sensitivity of the prediction model on 79 participants with overweight or obesity following two isocaloric (aerobic) exercise training programs known to significantly induce adipose tissue loss^43^. For this reason, we did not distinguish the effects of the two modalities in the statistical analysis.

Specifically, participants were installed on an electronically braked cycle ergometer (Corival, Lode B.V., Groningen, Netherlands) during all exercise training sessions. They were randomized into two groups: MICT (n = 32), in which workload was adjusted to 50% of peak power output, and HIIT (n = 47), in which participants performed 45 min of intermittent exercise consisting of 22 bouts of 1-min cycling at 100% of peak power output interspaced by 1-min passive recovery.

### Statistical analysis

Multiple linear regression analysis was performed to assess the relation between the measured AT or ATFM by the 41 slices and those predicted by the three single slices. The R^2^ was adjusted for the number of predictors. We used an automated variable selection procedure for forward, backward, and stepwise variable selection using the AIC (Akaike information criterion) estimator which evaluates the quality of each model, relative to each of the other models^44^. The Cook’s distance has been used for judging the influence of each observation on our regression models^45^: two outliers were removed. Assumptions of independence, normality and homogeneity of residuals have been satisfied with respective Durbin-Watson’s test, Shapiro–Wilk's test and Batlett’s test. Comparison between the predicted and measured AT and ATFM was performed using *t*-tests. Lin’s concordance coefficient (CCC, ranges from − 1 to 1) allowed to get a detailed impression of the degree of concordance^46,47^ between predicted and measured AT and ATFM. Levels closer to 1 indicate better agreement between methods. Bland and Altman plots^48^ were used to visualize, at an individual level, and assess the agreement^46^ between predicted and measured body composition as the bias ± random error.

Sensitivity of the prediction models to an exercise training program was also studied. Data were statistically analyzed using the nonparametric test Wilcoxon to compare the variations between the MRI reference method and the predictive method before (T0) and after 2-months (T2) of exercise training program.

The level of significance was set at p < 0.05 for all statistical analysis. The statistical software R version 3.6.0 was used for statistical analysis.

## Results

### Prediction of total AT

The 3 single MRI slices (at T6-T7, L4-L5 and mid-thigh), sex, age, weight and height of participants were included in linear regression models as independent variables, with total AT as the dependent variable (Table 2). Without height, predicted AT was more strongly correlated with measured AT with an adjusted R^2^ of 97.2 (p < 0.001) and the lowest AIC (504.11) (Table 2). Therefore, the best fit equation for total AT was: − 12.74105 + (0.02919 × age) + (4.27634 × sex (M = 0, F = 1)) + (0.22008 × weight) + (26.92234 × AT T6-T7) + (23.70142 × AT L4-L5) + (37.94739 × AT mid-thigh). There was no significant difference between predicted and measured AT (measured: 32.38 ± 13.27 kg, predicted: 32.38 ± 13.27 kg; p = 0.99).

**Table 2:** Regression coefficients of predictive equations (Eq) for adipose tissue (AT) and lean mass (ATFM).

Predictive equations for AT												
Eq	Constant	Age	Gender (M = 0, F = 1)	Height	Weight	AT T6-T7	AT L4-L5	AT mid-thigh	R^2^ (%)	Adjusted R^2^ (%)	AIC	p-value
1	0.5307	–	–	–	–	31.8244***	37.5211***	52.5211***	95.54	95.49	646.13	2.2^–16^
2	− 9.62934*	0.02707*	4.19641***	− 2.03987	0.22902***	26.52139***	37.35266***	37.35266***	97.24	97.17	505.55	2.2^–16^
3§	− 12.74105***	0.02919*	4.24634***	–	0.22008***	26.92234***	37.94739***	37.94739***	97.23	97.18	504.11	2.2^–16^

Predictive equations for ATFM												
Eq	Constant	Age	Gender (M = 0, F = 1)	Height	Weight	ATFM T6-T7	ATFM L4-L5	ATFM mid-thigh	R^2^ (%)	Adjusted R^2^ (%)	AIC	p-value
1	0.4215	–	–	–	–	27.9032***	37.7959***	86.4435***	87.14	87.02	816.31	2.2^–16^
2§	− 33.10721***	− 0.02363	3.58052***	30.02252***	0.08549***	11.36859***	27.82244***	58.62648***	92.66	92.49	650.62	2.2^–16^
3	-36.05863***	–	3.39693***	31.15260***	0.08614***	10.63188***	26.92911***	59.94185***	92.61	92.46	650.68	2.2^–16^

*Correlation coefficient was significant at p < 0.05.

***Correlation coefficient was significant at p < 0.001.

§ Best fit equation.

**Figure 1:**
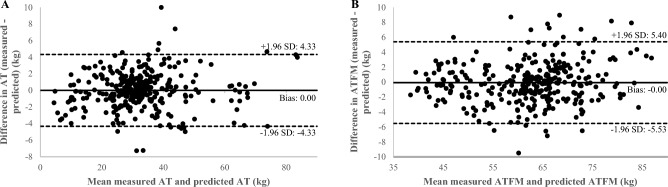
Bland–Altman plot agreement of adipose tissue (AT) with mean difference (solid line) and random error lines representing 95% limits of agreement (dashed lines) represent the comparison between predicted and measured AT in total sample (people with overweight/obesity or not: n = 310) (**A**). Bland–Altman plot agreement of adipose tissue free mass (ATFM) with mean difference (solid line) and random error lines representing 95% limits of agreement (dashed lines) represent the comparison between predicted and measured ATFM in total sample (people with overweight/obesity or not: n = 310) (**B**).

The predicted and measured methods showed high concordance (CCC = 0.986, Table 3). Figure 1A represents the Bland–Altman plot, showing the vast majority of data points were within the 95% limit of agreement, a random error of 4.3 kg and a bias toward zero (0.0006 ± 4.33 kg).

**Table 3:** Concordance between predicted and measured adipose tissue (AT) and adipose tissue free mass (ATFM).

	n	Lin’s coefficient		95% Confident interval	
		AT	ATFM	AT	ATFM
All participants	310	0.986	0.962	(0.982–0.989)	(0.953–0.969)
All women	70	0.987	0.951	(0.979–0.992)	(0.922–0.969)
All men	240	0.984	0.918	(0.979–0.987)	(0.896–0.935)
Overweight participants	93	0.859	0.942	(0.795–0.904)	(0.916–0.961)
Overweight women	14	0.875	0.694	(0.775–0.932)	(0.333–0.877)
Overweight men	79	0.834	0.852	(0.752–0.890)	(0.788–0.897)
Participants with obesity	179	0.980	0.964	(0.974–0.985)	(0.952–0.973)
Women with obesity	47	0.972	0.940	(0.952–0.984)	(0.895–0.966)
Men with obesity	132	0.981	0.912	(0.973–0.986)	(0.879–0.937)

**Figure 2:**
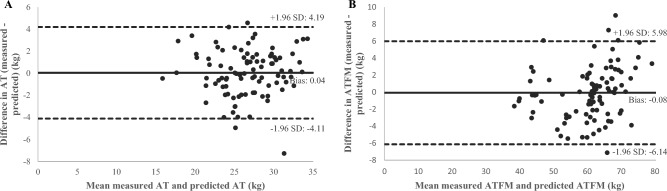
Bland–Altman plot agreement of adipose tissue (AT) (**A**) and adipose tissue free mass (ATFM) (**B**) in overweight people. Mean difference (solid line) and random error lines representing 95% limits of agreement (dashed lines) represent the comparison between predicted and measured methods.

**Figure 3:**
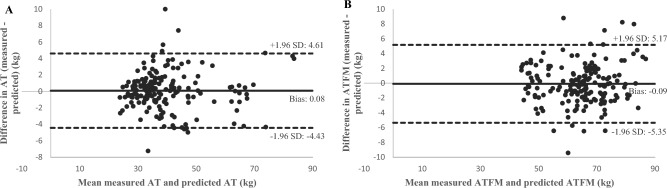
Bland–Altman plot agreement of adipose tissue (AT) (**A**) and adipose tissue free mass (ATFM) (**B**) in people with obesity. Mean difference (solid line) and random error lines representing 95% limits of agreement (dashed lines) represent the comparison between predicted and measured methods.

For women and men with overweight, Table 3 shows a good concordance between the predicted and the measured ones (women: CCC = 0.875, men: CCC = 0.834). For women and men with obesity, Table 3 shows a good concordance between the predicted and the measured values (women: CCC = 972, men: CCC = 0.981). Bland–Altman plots illustrate the difference between predicted and measured AT in people with overweight (total: 0.04 ± 4.15 kg, women: -0.26 ± 3.23 kg, men: 0.10 ± 4.3 kg) (Fig. 2A) and with obesity (total: 0.08 ± 4.52 kg, women: 0.23 ± 5.81 kg, men: 0.03 ± 3.98 kg) (Fig. 3A).

### Prediction of total ATFM

The same analysis was applied to the dependent variable total ATFM. Multiple regression equations for estimation of total ATFM were developed (Table 2). The best fit predictive regression equation included age, sex, weight, height and the 3 single MRI slices − 33.10721 + (− 0.02363 × age) + (− 3.58052 × sex (M = 0, F = 1)) + (30.02252 × height) + (0.08549 × weight) + (11.36859 × ATFM T6-T7) + (27.82244 × ATFM L4-L5) + (58.62648 × ATFM mid-thigh) (adjusted R^2^ = 92.5%; p < 0.001) (AIC = 650.62, Table 2). There was no significant difference between predicted and measured ATFM (measured: 62.26 ± 10.29 kg, predicted: 62.26 ± 9.90 kg; p = 0.99).

The predicted and measured methods showed high concordance (CCC = 0.962, Table 3). Measurement differences between the two methods were primarily within the 95% limit of agreement on the Bland–Altman plot (Fig. 1B). The two methods had an agreement with a bias tending towards zero (− 0.06 ± 5.46 kg).

Table 3 shows the concordance between predicted and measured, values was superior in men with overweight than in women with overweight (men: CCC = 0.852, vs. women: CCC = 0.694). For women and men with obesity however, the concordance was good (Table 3: women: CCC = 940, men: CCC = 0.912). Bland–Altman plots illustrate the difference between predicted and measured ATFM in overweight participants (total: − 0.08 ± 6.06 kg, women: 0.15 ± 4.71 kg, men: − 0.20 ± 6.29 kg) (Fig. 2B) and with obesity (total: − 0.09 ± 5.56 kg, women: − 0.05 ± 4.48 kg, men: − 0.19 ± 5.52 kg) (Fig. 3B).

### Sensitivity of the prediction models to an exercise training program

The measured and predicted AT after 2-months (T2) of exercise training did not significantly differ compared to baseline (T0) (T2: measured: 34.38 ± 10.82 kg, predicted: 34.42 ± 10.76 kg, p = 0.98; vs. T0: measured: 34.63 ± 11.14 kg, predicted: 33.41 ± 10.41 kg, p = 0.48). The same result was observed for ATFM variation (T2: measured: 62.61 ± 10.23 kg, predicted: 62.43 ± 9.96 kg, p = 0.91; vs. T0: measured: 62.37 ± 10.59 kg, predicted: 63.38 ± 9.15 kg, p = 0.52).

There was no significant difference between methods in AT variations (T2-T0 measured: − 0.24 ± 1.83 kg, p = 0.89; T2-T0 predicted: 1.01 ± 2.51 kg, p = 0.55) and in ATFM variations (T2-T0 measured: 0.24 ± 2.09 kg, p = 0.89; T2-T0 predicted: − 0.94 ± 2.20 kg, p = 0.54) induced by the exercise training program.

## Discussion

We developed and validated predictive models for AT and ATFM by using three single MRI slices (at T6-T7, L4-L5 and mid-thigh), sex, age, height and weight in order to facilitate the clinical assessment and follow-up of people with overweight/obesity.

Our results showed that the best fit models that we developed had a high adjusted R^2^ (AT: 97.2, ATFM: 92.5) and a low standard error of estimate (AT: 2.18 kg, ATFM: 2.69 kg). The SEE of our predictive equation compared quite well with that used by Schweitzer et al.^27^ in normal weight subjects (BMI: 25.3 ± 5.9 kg.m^−2^) despite a different involved population. Indeed, in the present study we built our equation in a population with obesity (272 subjects with overweight or obesity out of 310). In an attempt to predict total subcutaneous adipose tissue with a single slice at L3, Schweitzer et al.^27^ found lower R^2^ (between 0.92 and 0.90 for women and men respectively) and similar SEE (between 2.44 and 4.44 L) compared to ours (R^2^ = 97.2; SEE = 4.18 L = 2.18 kg/0.92 kg.L^−1^). Regarding ATFM, they reported R^2^ between 0.69 and 0.74 much lower than what we found (0.93) despite SEE slightly (between 1.93 L and 2.03 L) lower than ours (2.79 L assuming a constant density of 1.04 .L^−1^ for lean tissue (kg); these results are in line with those previously reported by Lee et al.^38^. These authors investigated total skeletal muscle estimates from single slice areas at the mid-thigh (244 normal weight subjects and 80 subjects obese with obesity) including anthropometric measures and reported that the final predictive model had a R^2^ = 0.91 and SEE = 2.2 kg. Overall, despite greater SEE for ATFM, our equations seem better than those reported in literature and our model represents a breakthrough in the rapid estimation of body composition by MRI in overweight and obesity.

Our results also showed a high concordance between predicted and measured methods (AT: CCC = 0.986, ATFM: CCC = 0.962, Table 3), a bias close to 0 both for AT and ATFM with moderate limits of agreement (Fig. 1A,B). This is a progress compared to other simplified approaches of body composition such as anthropometrics. Indeed, Lee et al.^38^ found higher bias (≈ 0.5 ± 6.5 kg) than us (ATFM: − 0.06 ± 5.46 kg). In addition, when the predictive model of Lee et al. was cross-validated including persons with obesity, there was a significant difference between measured and predicted ATFM values (− 2.3 ± 3.3 kg; p < 0.0001). According to the authors, this could be due to the fact that inter- and intra-muscular adipose tissue could not be distinguished by anthropometric measurements (including skinfold thickness)^38^. Hence, the prediction of body composition from MRI single slices seems more appropriate and accurate.

Furthermore, when this model was applied in women/men and overweight/obese subgroups, our results showed very good concordance for predicted AT (CCC: 0.834–0.987) and ATFM (CCC: 0.694–0.964) (Table 3), and a low bias (Fig. 2). Therefore, it seems that the best-fit equations can be used in a range of different profiles to quickly and accurately analyze body composition. However, in the overweight women subgroups (n = 14), the 95% confidence interval was large (0.333–0.877). A larger sample size in this subgroup might have provided higher concordance between predicted and measured methods.

There are several possibilities to explain the good concordance and agreement in these subgroups, including, the representativeness of the different types of obesity and the choice of three area slices. First, the present study was carried out in a large sample of people with and without obesity (n = 310, 70 women and 240 men) and hence variability in BMI (31.3 kg.m^−2^ ± 5.61 kg.m^−2^). The included participants also varied in age (50.8 ± 10.6 years) similar to the population in Lee et al.^39^ (i.e. range: 20–81, 39.6 ± 13.8 years). Despite the heterogeneity of the studied population, the best-fit models that we developed gave a good prediction of AT and ATFM in both men and women and in varying BMI (between 25 kg.m^−2^ and more). Secondly, over the last few years a single abdominal slice has been proposed to predict total fat and lean masses^27^ and to quickly assess subcutaneous adipose tissue and visceral adipose tissue^23–27,31^, which provides complementary information regarding cardiometabolic risk. One of the major drawbacks of this method was that a single slice cannot take into account the interindividual morphological differences (e.g., android or gynoid obesity)^33^. Accordingly, three single slices (at T6-T7, L4-L5 and mid-thigh) seems to be a good compromise between the time to analyze (20 min) and the accuracy of body composition prediction.

Recently, other localizations have been proposed to assess subcutaneous adipose tissue and visceral adipose tissue particularly around L3^25–28^ while L4-L5 was used in the present study. Maislin et al. (n = 668; BMI: 32.5 ± 4.9 kg.m^−2^)^25^ and Schaudinn et al. (n = 197; BMI: 33.8 ± 3.5 kg.m^−2^)^26^ concluded that the L2-L3 slice was better than L4-L5 slice to predict total visceral adipose tissue volume. This localization could decrease the standard error and improve the precision of our models. However, this remains to be tested. The mid-thigh has also been described as an optimal slice area to assess total ATFM^27^. In fact, the legs contain more than 50% of total body ATFM, predominating at the thigh^37^. Such a measurement would be for instance relevant to detect sarcopenic obesity. To the best of our knowledge, no study has investigated one common localization to assess AT and ATFM in the thoracic area. Hence, the single MRI slice at T6-T7 has been arbitrarily chosen hoping that it will the best site to determine, especially the mammary fat^35^. However, the localization of these three slices does not allow for the evaluation of fat in the gluteo-femoral region. This region is an area in which women with obesity often accumulate adipose tissue^21,33,34^. Thus, adding an MRI slice below the iliac crest could be relevant.

Whatever the method (measured or predictive), no significant modification of body composition was induced by 2 months of exercise training on ergocycle. A meta-analysis of Batacan et al.^43^ reported no systematic positive effect of body composition with a short-term exercise training program (< 3 months) in overweight or obese people. In the present study, we cannot definitely conclude on the potential sensitivity of our prediction models because we did not observe a modification of body composition. However, there was no significant difference between measured or predictive method after 2-months of intervention indicating at least that the 2 measurement methods are not discordant. Of note, estimating body composition with a unique slice (at L3 level) failed to track changes of body composition probably in persons losing weight because of lack of specificity of this slice^27^. Finally, it could be interesting to extend our exercise program (≥ 3 months)^49,50^ or to include a dietary intervention in order to induce a greater decrease in adipose tissue^50^ favoring the follow-up in clinical research setting.

The choice of a body composition method depends on the accuracy and precision needed. However, the acquisition time is also important to allow its use in a routine clinical setting as well as for research purposes. Indeed, a long acquisition period can be an obstacle to both the routine clinical research and patient’s comfort, in particular in an MRI scanner bore. A strength of the present study is to have demonstrated the ability of two developed equations to quickly estimate whole-body AT and ATFM using only three MRI slices for overweight or obese people with overweight/obesity. With the predictive method, the time required to collect (10 min) and to analyze (10 min) MRI images is well below the reference MRI method (20 min vs. 2 h). The associated gain in time and the reduced cost of this evaluation are crucial in a clinical research setting.

The choice of the methods also depends on the target population. For example, our model may not be generalizable to different patient populations who have specific body composition (e.g. COPD or highly trained athletes). For that reason, other predictive models must be developed for each specific population.

Since the main aim of the present study was to develop a simple tool to assess and follow adipose tissue and adipose tissue fat free mass in a French population including mainly persons with overweight/obesity (see Table 1 of the manuscript), it gave no insight into different phenotypes of obesity. Of course, an interesting perspective, could be to use the adipose tissue free mass and adipose tissue of the different slices locations (T6-T7, L4-L5 and at mid-thigh) to better characterize the various profiles of obesity (android, gynoid) and the associated cardiovascular risk. However, such a perspective could not be envisaged at the present condition since we did not measure the biological markers of this cardiovascular risk.

In conclusion, predictive equations with three MRI slices (T6-T7, L4-L5 and mid-thigh) were effective to quickly and accurately assess the body composition of people with overweight or obesity compared to the reference methods.

The findings we herein report have thus the potential to contribute to a fast and reliable estimation of AT and ATFM in overweight or obesity in clinical routine.

## Data Availability

The datasets generated during and/or analysed during the current study are available from the corresponding author on request.
